# Supplementary figures and images for: Comparative transcriptome analysis revealed genes involved in the fruiting body development of Ophiocordyceps sinensis
Source: PeerJ. 2020 Jan 16;8:e8379. doi: 10.7717/peerj.8379 (PMC6970007; doi:10.7717/peerj.8379)

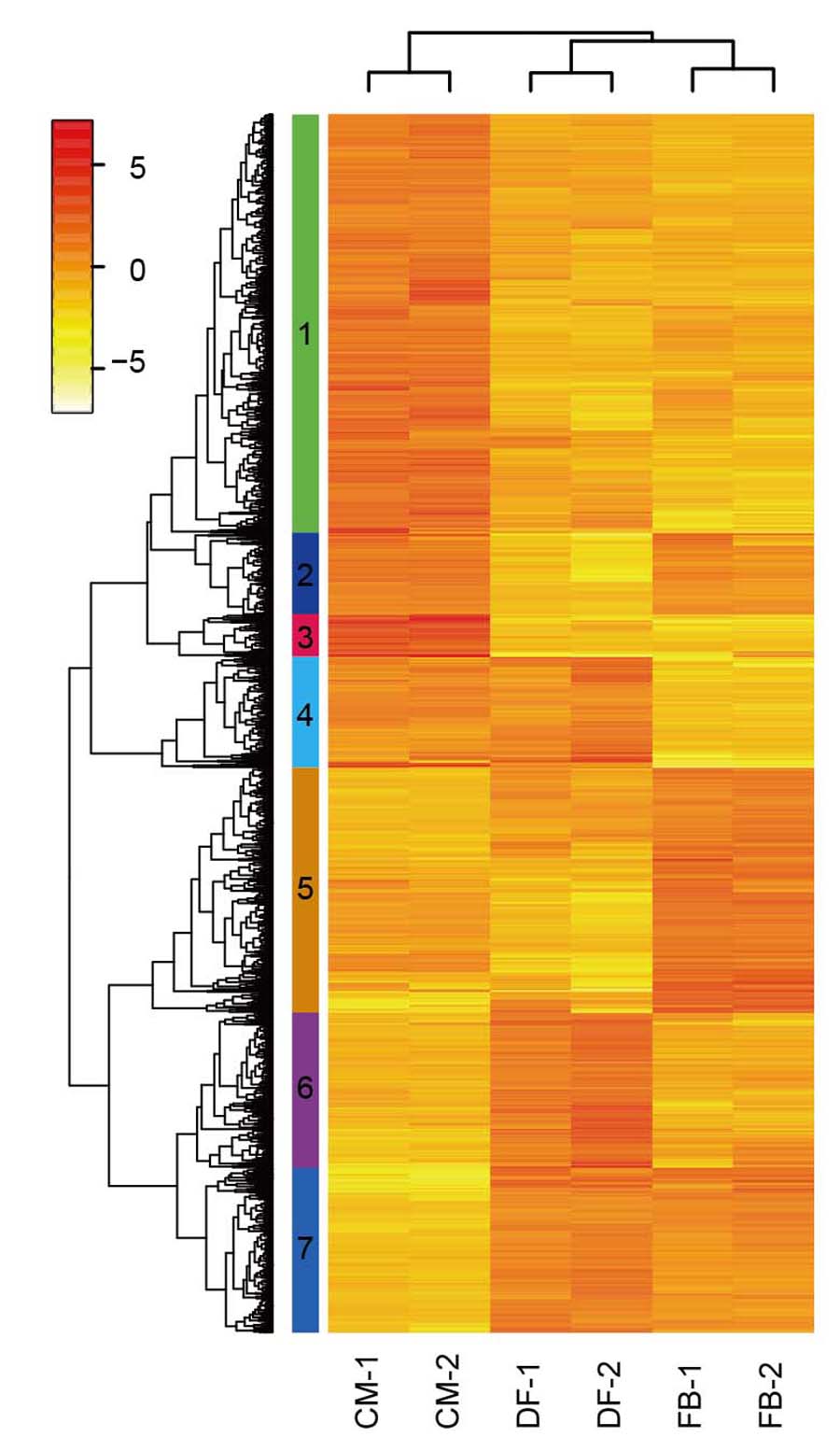

Supplement: Supplemental Information 5 [file peerj-08-8379-s005.jpg]
