# Supplementary material for: Comparative transcriptome analysis revealed genes involved in the fruiting body development of Ophiocordyceps sinensis
Source: PeerJ. 2020 Jan 16;8:e8379. doi: 10.7717/peerj.8379 (PMC6970007; doi:10.7717/peerj.8379)
Supplement: Supplemental Information 11 [file peerj-08-8379-s011.docx]

|  | **Seq ID** | **Primer F** | **PPrimer R** |
| --- | --- | --- | --- |
|  |  |  |  |
| 1 | MSTRG.5600 | 5’-AGGCTAACTGCTCCTTGCTGTC -3’ | 5’-GCAGAGCGTTGATTTCCTCCCA -3’ |
| 2 | MSTRG.7313 | 5’-ACGCTGTGGGCTAATACATCTCC-3’ | 5’-GGATGGTACTGTCGCGGCAT-3’ |
| 3 | MSTRG.3832 | 5’-CGGCTACTTCGACCTCCTCATC-3’ | 5’-GACAATGGCGCGGATGATCTG-3’ |
| 4 | MSTRG.6014 | 5’-GTGAACACGGCACCGTCAAG-3’ | 5’-CCTCTTATTGGTCGTCTGGTGCC-3’ |
| 5 | MSTRG.5638 | 5’-TGAAGCTGCGTGTTGCTGAC-3’ | 5’-CACCAGGACAAGTCGGTCGT-3’ |
| 6 | MSTRG.2865 | 5’-TCGCACCTCGAAGCAATCAATCAC-3’ | 5’-GTGTGCTCGCTGTATTCGTAAGCT-3’ |
| 7 | MSTRG.2218 | 5’-AGACATGGAAGCCGACGCTGATC-3’ | 5’-CGTCGATTCAAGGCGGTTGGA-3’ |
| 8 | MSTRG.5297 | 5’-CAAGGACTGGAGCAAGAGCGATC -3’ | 5’-GAAACGCCACGGACTCAGCT -3’ |
| 9 | MSTRG.5396 | 5’-CGGCATAGGCTTCGGCATCAAGATC -3’ | 5’-GTGGCAAACTTGAGCGAGCGGT -3’ |
| 10 | MSTRG.8670 | 5’-TCATCATGCGTGGCGTCGAG-3’ | 5’-GATCTTGTTGAAGGCGTTTGTGGCG-3’ |
